# Long noncoding RNA LINC01559 promotes pancreatic cancer progression by acting as a competing endogenous RNA of miR-1343-3p to upregulate RAF1 expression

**DOI:** 10.18632/aging.103487

**Published:** 2020-07-17

**Authors:** Xiao Chen, Jie Wang, Fei Xie, Tinggang Mou, Pingyong Zhong, Hao Hua, Pan Liu, Qin Yang

**Affiliations:** 1 Department of Osteology, The First Peoples Hospital of Neijiang, Neijiang, Sichuan, China; 2 Department of Hepatic-Biliary-Pancreatic Surgery, The First Peoples Hospital of Neijiang, Neijiang, Sichuan, China; 3 Department of Gastroenterology, The First Peoples Hospital of Neijiang, Neijiang, Sichuan, China

**Keywords:** LINC01559, miR-1343-3p, RAF1, pancreatic cancer

## Abstract

Background: An increasing number of studies have shown that lncRNAs are involved in the biological processes of pancreatic cancer (PC). Hence, we investigated the role of a novel noncoding RNA, LINC01559, involved in PC progression.

Results: LINC01559 and RAF1 were highly expressed in PC, while miR-1343-3p had low expression. High expression of LINC01559 was significantly associated with large tumors, lymph node metastasis, and poor prognosis. Functional experiment results revealed that silencing of LINC01559 significantly suppressed PC cell proliferation and metastasis. Meanwhile, LINC01559 could act as a ceRNA to competitively sponge miR-1343-3p to up-regulate RAF1 and activate its downstream ERK pathway

Conclusions: LINC01559 functions as an oncogene in PC progression through acting as a ceRNA of miR-1343-3p. Hence, LINC01559 is a potential diagnostic and therapeutic target.

Methods: RT-qPCR was performed to determine the expression of LINC01559 and miR-1343-3p in PC. Individual patient data were collected to investigate the correlation between clinicopathological features and LINC01559 expression. Subsequently, the expression of LINC01559, miR-1343-3p, and RAF1 was altered using transfection of vectors or inhibitors. Gain- and loss-of-function assays and mechanistic assays were applied to verify the effects of LINC01559, miR-1343-3p, and RAF1 on PC cell proliferation and metastasis *in vivo* and *in vitro.*

## INTRODUCTION

Pancreatic cancer (PC) is one of the most common digestive malignant tumors, with a 5-year survival rate lower than 10%, making it a major carcinogenic factor in human morbidity and mortality worldwide [1]. The absence of typical symptoms at an early stage, rapid disease progression, and lack of effective treatment strategies are the prominent clinical characteristics of pancreatic cancer, leading to poor patient prognosis [2]. Surgery is the most effective treatment for pancreatic cancer, but because of the insidious onset of this disease, most patients are diagnosed at an advanced stage when radical resection is no longer possible [3]. Unfortunately for these patients, adjuvant chemotherapy is limited in terms of its type and efficacy [4]. Therefore, to improve the prognosis of patients with PC, there is an urgent need to identify new diagnostic biomarkers and develop innovative molecular therapeutic strategies.

Accumulating evidence has indicated that the dysregulation of long noncoding RNAs (lncRNAs) is closely related to the progression and metastasis of multiple tumors including PC [5]. lncRNAs are defined as noncoding RNAs of more than 200 nucleotides in length, which lack the ability to directly encode proteins [6]. Researchers have demonstrated that lncRNAs are involved in multiple biological processes, such as cell proliferation, migration, immune regulation, transcriptional modification, and drug resistance [7]. Increasing evidence has revealed that lncRNAs act as vital regulators in the carcinogenesis and progression of pancreatic cancer, and that they regulate gene expression by various mechanisms, one of which is acting as sponges of microRNAs (miRNAs) [8, 9]. For example, previous studies suggested that the lncRNA LUCAT1 acts as a molecular sponge of miR-539, which inhibits cell proliferation and cell cycle progression in PC [10]. In addition, the lncRNA SBF2-AS1 was shown to be involved in gemcitabine resistance in PC and the underlying mechanism was that SBF2-AS1 acted as a competing endogenous RNA (ceRNA) to sponge miR-142-3p to suppress the expression of miR-142-3p and counteract the inhibition of TWF1, which could modulate drug sensitivity [11].

miRNAs are small noncoding RNAs of about 19-24 nucleotides in length that play a regulatory role in cells by directly binding to the 3′-untranslated region (3′-UTR) of target mRNAs, leading to RNA degradation or post-transcriptional inhibition [12]. A large number of studies have proved that miRNAs play important roles in tumor development, such as in cell proliferation, apoptosis, autophagy, and metastasis [13]. miRNAs can act as both oncogenes and tumor suppressors according to the function of their target mRNA [14]. A previous study proved that miR-1343-3p exhibits low expression in PC, but its function remained unclear.

In the present study, we focused on LINC01559 and aimed to identify its biological function in PC progression and the mechanism involved. We found that LINC01559 was overexpressed in PC cells and tissues. Furthermore, we found that LINC01559 acted as an oncogenic RNA to promote cell proliferation and metastasis, and could suppress the expression of miR-1343-3p by functioning as a sponge of it, leading to the increased expression of the tumor promoter RAF1 in PC. Overall, our study revealed that this LINC01559/miR-1343-3p/RAF1 pathway has potential diagnostic and therapeutic value for PC.

## RESULTS

### lncRNA LINC01559 was significantly overexpressed in PC tissues and cell lines

**Figure 1:**
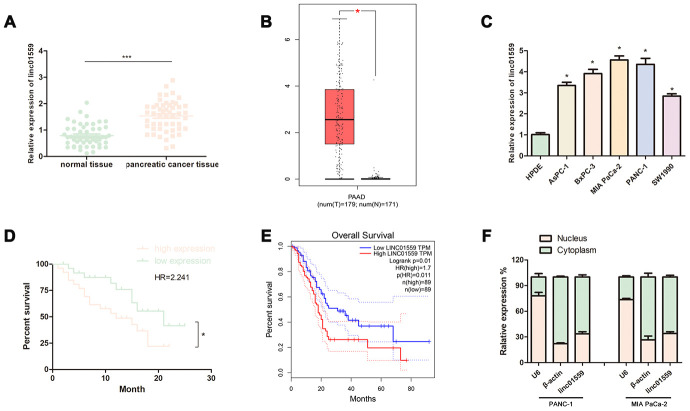
**LncRNA LINC01559 was overexpressed in PC tissues and cells and was clinically relevant in PC.** (**A**) RT-qPCR analysis of LINC01559 expression in PC tissues. (**B**) The expression of LINC01559 in PC tissues obtained from TCGA database. (**C**) A differential expression pattern of LINC01559 was observed in PC cell lines. (**D**) The survival curve of different expression of LINC01559 in PC. (**E**) The overall survival months in patients with PC obtained from TCGA database. (**F**) The relative expression of LINC01559 in nucleus and cytoplasm. All experiments were performed three times and data were presented as mean ± SD. *p < 0.05, ***p < 0.001

To investigate whether the lncRNA LINC01559 was associated with PC progression, we determined its expression patterns via RT-qPCR. Fifty-one pairs of cancerous tissues and adjacent healthy tissues were collected for parallel PCR testing; the results revealed that LINC01559 expression in the cancerous tissues was significantly higher than that in the paired adjacent normal tissues (Figure 1A). Furthermore, the TCGA database further indicated that LINC01559 was highly expressed in PC tissues (Figure 1B). To verify the expression of LINC01559 in cell lines, five PC cell lines were used to test the relative LINC01559 expression compared with that in HPDE. The results suggested that LINC01559 was overexpressed in the PC cell lines (Figure 1C). The correlation between clinicopathological characteristics and LINC01559 is shown in Table 1. The results indicated that high expression of LINC01559 was significantly associated with large tumors and lymph node metastasis. Notably, combined with the follow-up information of the patients, Kaplan–Meier analysis was applied to create a survival curve of overall survival. The results illustrated that patients with high LINC01559 expression had poorer prognosis than patients with its low expression (Figure 1D). Meanwhile, the data from the TCGA database had similar results that highly expressed LINC001559 led to poorer prognosis. (Figure 1E). The analyses of RNA extracted from the nucleus and cytoplasm demonstrated that LINC01559 was mainly located in the cytoplasm (Figure 1F).

**Table 1:** General clinicopathological characteristics of patients.

**Clinical Epidemiology and Clinicopathologic Feature**		**LINC01559**		***p value***
		**low expression**	**high expression**	
all cases		25	26	
age				
	≤50	11	14	0.5793
	>50	14	12
gender				
	male	13	9	0.2640
	female	12	17
diameter of tumor				
	≤2	18	9	0.0115
	>2	7	17
pathological grading				
	I/II	10	8	0.5653
	III/IV	15	18
lymphatic metastasis				
	Negative	19	10	0.0107
	Positive	6	16
distant metastasis				
	Negative	22	20	0.4654
	Positive	3	6
TNM stage				
	I/II	18	21	0.5230
	III/IV	7	5

### Silencing of LINC01559 expression suppressed PC cell proliferation in vivo and in vitro

**Figure 2:**
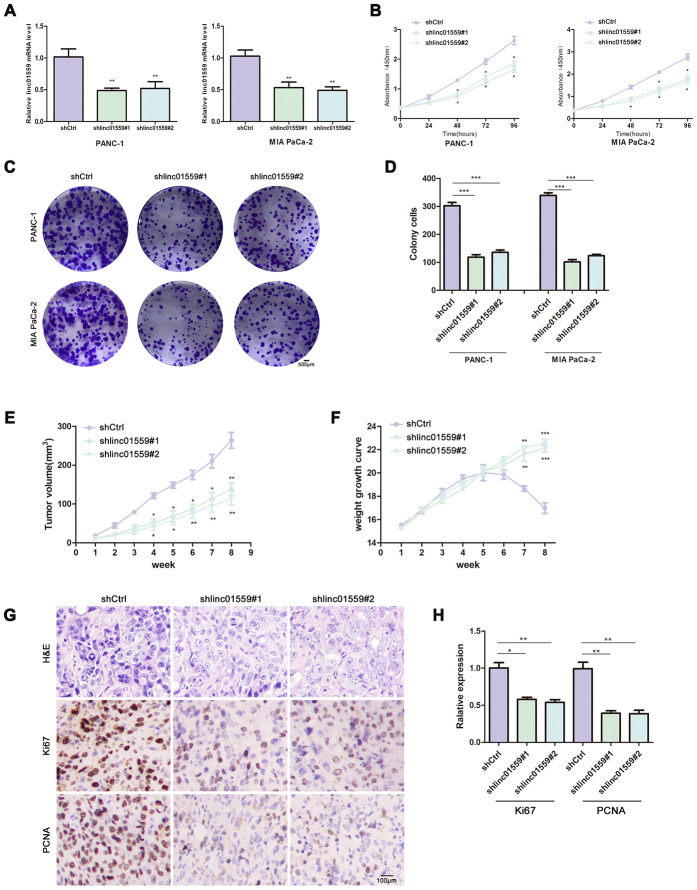
**Silence of LINC01559 expression suppressed PC cell proliferation in vivo and in vitro.** (**A**) The effects of transfection in PC cell lines were detected by PCR assay. (**B**) CCK-8 assay was performed to test the cell viability and proliferation in control group (shCtrl) and silence groups (shlinc01559). (**C**, **D**) Colony formation assay was performed to test the cell colony ability in shCtrl and shlinc01559. (**E**) Tumor volume of the subcutaneous xenografts in shCtrl and shlinc01559. (**F**) Weight change curve. (**G**) IHC staining for LINC01559 and representative images of three pairs of subcutaneous xenograft tissue (100×).(bar: 100 μm) (**H**) The relative expression of Ki67 and PCNA in tumor tissue. *p < 0.05, **p < 0.01, ***p < 0.001

To assess the effect of LINC01559 on proliferative ability, we first designed two different downregulated sequences and cloned them into lentivirus vector. Then, we successfully infected PANC-1 and MIA PaCa-2 PC cell lines with lentivirus vector and compared the effects of transfection with those for control vectors (Figure 2A). CCK-8 assay was carried out to investigate the cell proliferative ability. The results revealed that silencing of LINC01559 expression significantly inhibited cell viability (Figure 2B). In addition, colony formation assay was used to test the colony-forming ability of PC cells. The results illustrated that downregulation of LINC01559 expression suppressed colony formation compared with that in the shCtrl groups (Figure 2C, 2D). To further investigate the role of LINC01559 in PC tumorigenesis, we constructed a subcutaneous tumorigenesis model *in vivo*. The results from this model suggested that the silencing of LINC01559 expression induced slower growth of tumors and that the tumor volumes were significantly smaller than those in the control group (Figure 2E). Additionally, the mice in the control group began to lose weight after 6 weeks of feeding, possibly as a result of them being more prone to cachexia than the downregulated group (Figure 2F). Proliferation-related indexes Ki67 and PCNA were applied to detect the tumors of mice via IHC. The results proved that Ki67 and PCNA were more highly expressed in the shCtrl groups than in the expression-silenced groups (Figure 2G, 2H).

### Silencing of LINC01559 expression suppressed PC cell migration and invasion

**Figure 3:**
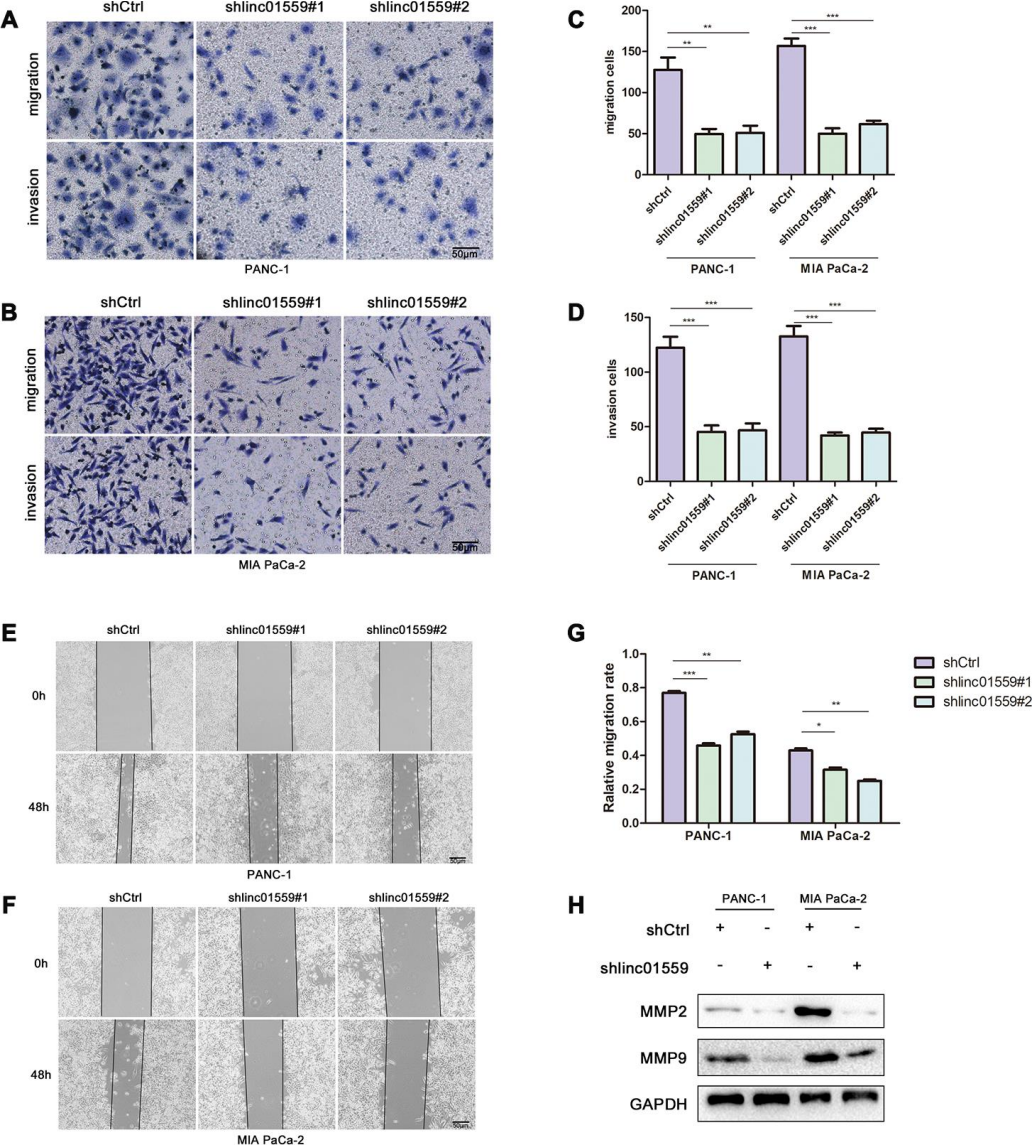
**Silence of LINC01559 expression suppressed PC cell migration and invasion.** (**A**–**D**) The effect of LINC01559 expression on migration and invasion of PC cells by using transwell assay (200×) (bar: 50 μm). (**E**–**G**) Wound healing assay demonstrates the suppressed migration ability of shlinc01559 compared to shCtrl in PANC-1 and MIA PaCa-2 cell lines (200×) (bar: 50 μm). (**H**) Western blotting was used to detect MMP2 and MMP9 in PC cells, which were indexs for migration ability and were reported in previous studies.*p < 0.05, **p < 0.01, ***p < 0.001

To further verify the hypothesis that LINC01559 promotes PC cell metastasis, we cultured transfected cells for a Transwell assay and wound healing assay to test their migration abilities. The results indicated that silencing of LINC01559 expression in PC cells led to significantly weaker migration and invasion abilities (Figure 3A–3G). The MMP2 and MMP9 proteins were shown to be closely associated with PC cell metastasis in previous studies [15], and thus we used western blotting assay to detect their expression. The results suggested that MMP2 and MMP9 had lower expression in the LINC01559-knockdown groups than in the control groups (Figure 3H).

### LINC01559 functioned as a ceRNA for miR-1343-3p

**Figure 4:**
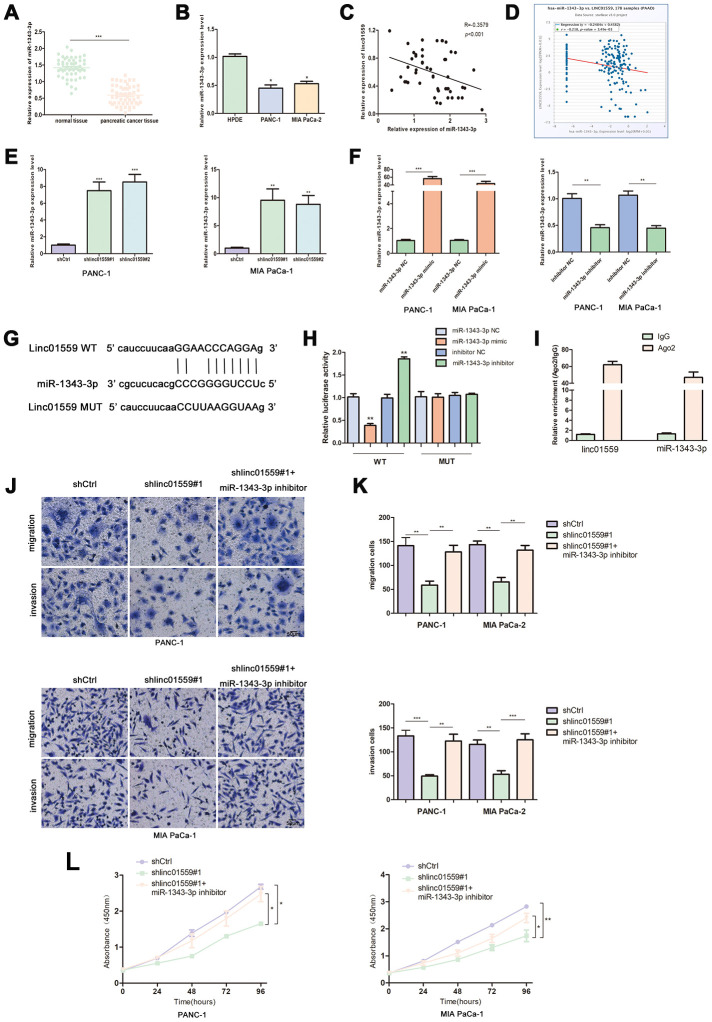
**LINC01559 functioned as a ceRNA for miR-1343-3p.** (**A**) RT-qPCR analysis of miR-1343-3p expression in PC tissues and cell lines (**B**). (**C**, **D**) Pearson correlation analysis was used to investigate the correlation between LINC01559 and miR-1343-3p from our data and TCGA. (**E**) miR-1343-3p expression in shCtrl or shlinc01559 groups. (**F**) The expression of miR-1343-3p was detected by PCR after transfecting miR-1343-3p mimics or inhibitors in PC cells. (**G**) The prediction binding site of miR-1343-3p in LINC01559 3’UTR. (**H**) Luciferase assay and (**I**) RIP assay were applied to investigate the direct interaction between LINC01559 and miR-1343-3p. (**J**–**L**) Functional rescue experiments was performed to verify the effect of miR-1343-3p inhibitor on migration and proliferation in shlinc01559 group. *p < 0.05, **p < 0.01, ***p < 0.001.

One of the most important mechanisms by which lncRNAs exert their functions is to act as ceRNAs to sponge target miRNAs. Therefore, we searched for and screened potential targets of LINC01559 via a network database analysis in Starbase 3.0. This led to miR-1343-3p being identified as a candidate gene for subsequent validation and research. RT-qPCR was performed to determine the expression patterns of miR-1343-3p; the results revealed that miR-1343-3p was expressed at a low level in PC tissues and cell lines compared with the case in normal tissues and HPDE (Figure 4A, 4B). The results of RT-qPCR analysis revealed that the expression of LINC01559 was negatively correlated with miR-1343-3p expression, which was supported by the results of the bioinformatic database from Starbase 3.0 (Figure 4C, 4D). Subsequently, miR-1343-3p expression in LINC01559-knockdown and control groups was determined by RT-qPCR. The results confirmed that miR-1343-3p was significantly highly expressed in the LINC01559-silenced groups compared with that in the control groups (Figure 4E). To further investigate whether miR-1343-3p was the target of LINC01559, we first transfected miR-1343-3p mimics, inhibitors, and their control into PC cell lines to verify the effects of transfection (Figure 4F). The predicted binding site of miR-1343-3p in the LINC01559 3′UTR is shown in Figure 4G. Luciferase reporter assay confirmed this interaction, showing that LINC01559 wild type expressed lower luciferase activity with miR-1343-3p upregulated and higher luciferase activity with miR-1343-3p inhibition; meanwhile, the LINC01559 mutant exhibited its normal expression (Figure 4H). RIP assay further confirmed the direct interaction between LINC01559 and miR-1343-3p, which were enriched in Ago2 complex (Figure 4I). The experiments on the rescue of cell function indicated that the inhibition of miR-1343-3p expression could partly rescue the inhibitory effects of downregulated LINC01559 on the migration, invasion, and proliferation abilities of PC cells (Figure 4J–4L).

### miR-1343 targeted RAF1 and LINC01559 positively regulated RAF1 via miR-1343-3p

**Figure 5:**
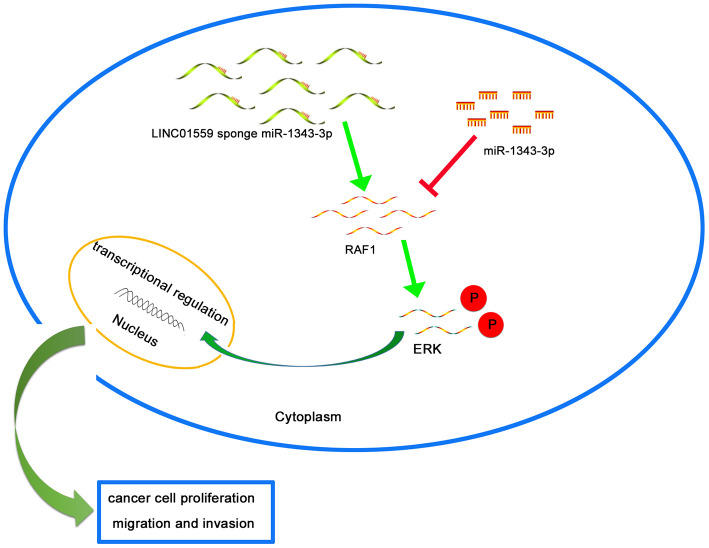
**MiR-1343 targeted RAF1 and LINC01559 positively regulated RAF1 via miR-1343-3p.** (**A**) The prediction binding site of miR-1343-3p in RAF1 3’UTR. (**B**) Luciferase assay was applied to investigate the direct interaction between RAF1 and miR-1343-3p. (**C**) The RAF1 mRNA expression in miR-1343-3p mimic and miR-1343-3p inhibitor groups. (**D**) The RAF1 mRNA expression in shCtrl and shlinc01559 groups. (**E**) The RAF1 protein expression in shCtrl and shlinc01559 groups. (**F**) The relative expression of RAF1 in nude mouse tumors. (**G**) The RAF1 mRNA expression after transfecting RAF1 vector or siRNA in PC cells. (**H**–**J**) Functional rescue experiments was performed to verify the effect of RAF1 vector on migration and proliferation in shlinc01559 group. (**J**) Western blotting showing the expression change of proteins involved in the ERK signaling pathway.

**Figure 6:**
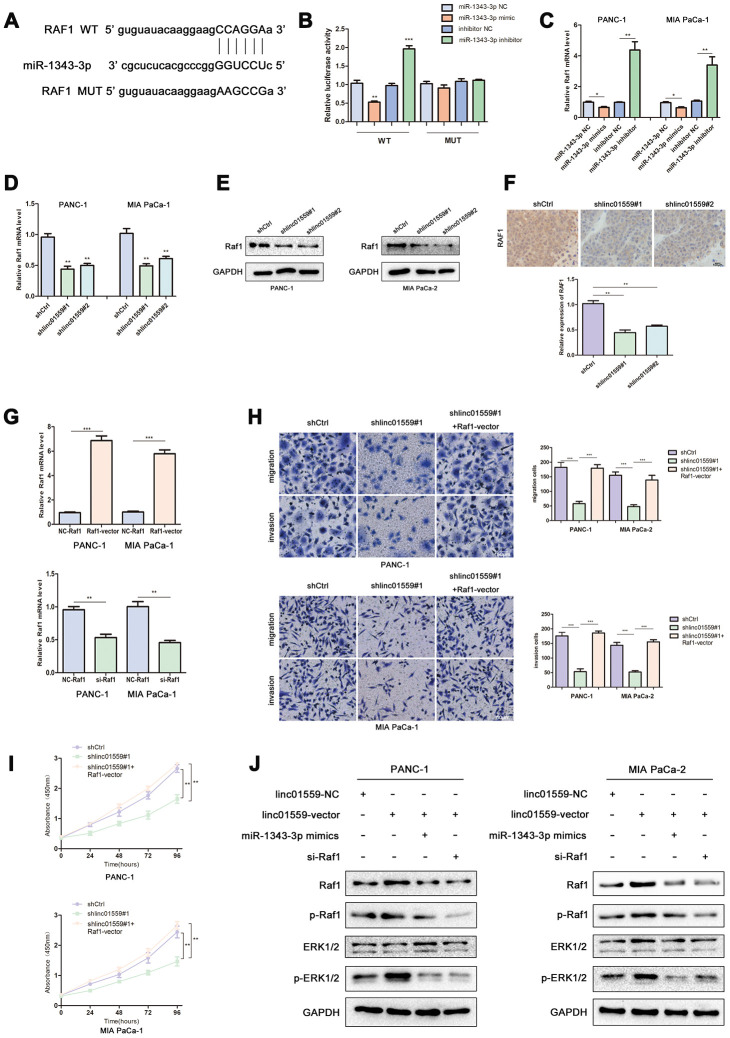
**Schematic diagram of mechanism on this research.** LINC01559 promotes RAF1-mediated proliferation and metastasis via decoying of miR-1343-3p in PC.

To further explore the mechanism underlying LINC01559’s promotion of migration and proliferation, we predicted RAF1 as a potential target through Starbase 3.0; the interacting sites of miR-1343-3p and RAF1 are displayed in Figure 5A. Luciferase reporter analysis confirmed the predicted results, suggesting that miR-1343-3p overexpression induced lower luciferase activity in RAF1 wild type, but miR-1343-3p knockdown induced higher luciferase activity in RAF1 wild type; meanwhile, RAF1 mutant exhibited normal expression (Figure 5B). RT-qPCR analysis confirmed that RAF1 expression was highly consistent with LINC01559 expression in PC cells, whereas RAF1 expression was negatively correlated with miR-1343-3p expression in PC cells (Figure 5C, 5D). To confirm that LINC01559 affected the protein expression of RAF1, western blotting assay was performed to confirm the connection, revealing that RAF1 was expressed at a low level in LINC01559-knockdown groups (Figure 5E). IHC assay was performed to detect the expression of RAF1 in mouse tumors, suggesting that RAF1 was more highly expressed in the control groups than in the silenced expression groups (Figure 5F). RT-PCR analysis indicated that PC cells were successfully transfected with RAF1 vector and si-RAF1 (Figure 5G). Functional experiments suggested that RAF1 overexpression could partly reverse the regulatory effects on cell proliferation, migration, and invasion induced by LINC01559 suppression (Figure 5H, 5I). Western blotting results revealed that LINC01559 overexpression was positively associated with RAF1 expression. In addition, phosphorylated RAF1 was also activated, leading to an increase in downstream ERK phosphorylation, but the total ERK did not change. This suggested the activation of the MAPK signaling pathway, which has been widely reported to be related to the proliferation and metastasis of pancreatic cancer. However, the upregulation of miR-1343-3p or knockdown of RAF1 expression could partly reverse the up-regulation of protein expression, such as phosphorylated RAF1 and phosphorylated ERK. (Figure 5J). This study demonstrated LINC01559 could promote RAF1-mediated proliferation and metastasis via decoying of miR-1343-3p in PC, a molecular mechanism diagram was shown in Figure 6.

## DISCUSSION

Recently, PC has been identified as one of the most common aggressive malignancies of the digestive system and has imposed enormous pressures on public health [16]. Considering the limitations of conventional methods for diagnosing and treating PC, it is necessary to find novel and effective diagnostic and therapeutic strategies. Accumulated evidence indicates that the dysregulation of lncRNAs plays an essential role in regulating pancreatic cancer progression. For example, Liu’s study revealed that the lncRNA FOXP-AS1 was highly expressed in PC and that its higher expression was significantly associated with a poorer prognosis compared with that of patients with lower FOX-AS1 expression [17]. In addition, the lncRNA SNHG14 was significantly correlated with advanced TNM stage in patients with PC. High expression of SNHG14 could interact with EZH2 to affect the expression of E-cadherin, a key protein of EMT, promoting PC cell migration [18]. In contrast, some lncRNAs also acted as tumor suppressor genes in PC, such as LINC00673, which was expressed at a significantly low level in PC cells and could regulate invasion and migration in PC via inhibiting miR-504 [19]. In addition, the lncRNA CASC2 served as a sponge to combine miR-24, which led to high expression of its downstream target MUC6, suppressing cell proliferation and progression in PC cells [20]. In our study, we selected LINC01559 as our target gene via analysis using a bioinformatic database. Previous studies suggested that LINC01559 could predict overall survival and recurrence of renal cell carcinoma patients [21]. RNA-sequencing was performed to identify novel lncRNAs in breast cancer, revealing that LINC01559 was significantly dysregulated in this disease context and might act as an oncogene [22]. Meanwhile, our results demonstrated that LINC01559 was highly expressed in PC tissues and cell lines, and it was significantly associated with poor prognosis. Meanwhile, in patients, higher LINC01559 expression was positively correlated with tumor size and lymphatic metastasis. PC cell proliferation and metastasis were clearly inhibited by silencing LINC01559 expression.

The dysfunction of miRNAs has been shown to be closely associated with tumorigenesis and progression in multiple tumors [23]. For example, miR-1343-3p was identified as a tumor suppressor in many cancers. In addition, TEAD4 was reported to be an oncogenic factor in gastric cancer and to be associated with a poor overall survival rate. Moreover, TEAD4 expression and activation were shown, in part, to be mediated by knockdown of the expression of miR-1343-3p [24]. miR-1343-3p was thus evaluated for its correlation with the clinicopathological features of lung adenocarcinoma, and its expression was found to be low in patients with vascular invasion [25]. Yuan’s study showed that miR-1343-3p was constantly downregulated in colon, prostate, and pancreatic cancers, including in early-stage colon cancer; in addition, miR-1343-3p could be used to differentiate pancreato-biliary malignancy from nonmalignant diseases [26]. In our study, LINC01559 was shown to potentially act as a ceRNA to bind with miR-1343-3p, inhibiting the latter’s expression and thereby promoting PC progression. Subsequently, functional rescue experiments confirmed that miR-1343-3p was the target of LINC01559 and acted as a tumor suppressor in PC.

Accumulating evidence has demonstrated that RAF1 is an important part of the classical pathway of RAF/MEK/ERK signaling and is highly expressed in multiple cancers including pancreatic cancer [27, 28]. RAF1 transferred from the cytoplasm to the membrane and was activated by tyrosine kinases; then, the phosphorylation of RAF1 activated downstream MEK and ERK and ultimately played a variety of biological roles. Phosphorylated ERK could enter the nucleus from the cytoplasm to participate in the regulation of certain transcription factors (such as c-Fos and c-Jun), resulting in cell proliferation and migration. The latest research has revealed that the combined removal of EGFR and RAF1 expression resulted in complete regression of PDAC tumors driven by Kras/Trp53 mutations in genetically engineered mice [29]. RAF1 was identified as a target of miR-1343-3p and its expression was positively correlated with LINC01559, promoting PC cell proliferation and invasion via activation of the ERK signaling pathway. In contrast, the silencing of RAF1 or upregulation of miR-1343-3p could partly reverse the effects of silencing LINC01559.

## CONCLUSION

In summary, we identified the oncogenic role of LINC01559 in proliferation and metastasis in PC. Additionally, we revealed that the mechanism underlying this might involve LINC01559 acting as a competing endogenous RNA of miR-1343-3p to up-regulate RAF1 expression, which would further activate the ERK signaling pathway. Therefore, LINC01559 might become a potential diagnostic and therapeutic target in PC.

## MATERIALS AND METHODS

### Collection of tissue samples

PC tissues and their paired adjacent normal tissues were obtained from 51 patients at the First People’s Hospital of Neijiang. No other treatment was performed before this study, and tissue samples collected after surgical resection from 2017 to 2019 were immediately stored at −80°C. The characteristics of the patients are summarized in Table 1. This study was approved by the Ethics Committee of the First People’s Hospital of Neijiang.

### Cell treatment and transfection

Human PC cell lines (AsPC-1, BxPC-3, PANC-1, MIA PaCa-2, SW1990) and a human normal pancreatic ductal epithelial cell line (HPDE) were purchased from West China Hospital of Sichuan University. HPDE, AsPC-1, and BxPC-3 were incubated in RPMI1640 (Hyclone, USA) with 10% FBS (Gibco, USA) at 37°C with 5% CO_2_. The rest of the cell lines were incubated under the same environmental conditions but in DMEM (Hyclone, USA).

The vectors encoding LINC01559 (linc01559-vector) and RAF1 (RAF1-vector), the negative control vector (linc01559-NC, NC-RAF1), RAF1 siRNA (si-RAF1), miRNA mimic (miR-1343-3p mimic), control mimic (miR-1343-3p NC), miRNA inhibitor (miR-1343-3p inhibitor), and control inhibitor (inhibitor NC) were purchased from Ruibo (Guangzhou, China). The lentiviruses encoding silenced LINC01559 (shlinc01559#1, shlinc01559#2) and control lentivirus (shCtrl) were purchased from GeneChem (Shanghai, China). Cell transfection or infection was carried out following the manufacturer’s protocol.

### Real-time quantitative polymerase chain reaction (RT-qPCR)

TRIzol reagent (Invitrogen, USA) was used to extract total RNA from PC tissues or cells following the manufacturer’s protocol. The ReverTra Ace qPCR RT Kit (Takara, China) was applied to reverse-transcribe total RNA into cDNA. The SYBR Green Realtime PCR Master Mix (Takara) was used for qPCR with cDNA as templates and GAPDH or U6 as internal controls. The relative expression of genes was calculated and analyzed through the 2^−ΔΔCt^ method. All PCR sequences involved in this study are detailed in Supplementary Table 1.

### Dual-luciferase reporter gene assay

The biological prediction website Starbase 3.0 was used to predict and analyze the target genes of miR-1343-3p. The wild-type (WT) or mutant (MUT) sequences of LINC01559 or RAF1 were cloned into the dual luciferase reporter vector pGL3. Lipofectamine 2000 (Invitrogen, USA) was applied when co-transfecting plasmids and miR-1343-3p mimic or miR-1343-3p NC into PANC-1. Furthermore, plasmids and miR-1343-3p inhibitor or inhibitor NC were co-transfected into PANC-1. At 48 h after transfection, the relative luciferase activity was determined in accordance with the instructions of the double luciferase reporter kit (Promega, USA).

### RIP assay

Magna RIP assay kit (Millipore, USA) was applied to investigate the interaction between our experimental targets, in accordance with the supplied protocol. PC cells were subjected to RIP lysis and exposed to magnetic beads conjugated with Ago2 or IgG antibodies (Sigma-Aldrich). RT-qPCR was performed to determine the expression of LINC01559 and miR-1343-3p from the immunoprecipitates.

### Transwell assay

Twenty-four-well Transwells with or without Matrigel precoating (1:8 ratio with DMEM) (BD, USA) were used to determine the invasion and migration abilities. Approximately 1×10^4^ cells per chamber in 200 μl of serum-free medium were placed into the upper chamber with or without Matrigel. The lower chamber was supplemented with 800 μl of DMEM with 20% fetal bovine serum. Then, the cells were cultured for 24–30 h. Subsequently, the upper chamber was washed with PBS and fixed with 4% paraformaldehyde, followed by staining with 1% crystal violet. An Olympus BX51 microscope was used to photograph the cell layers.

### CCK-8 assay

The same number of transfected PC cells were cultured into 96-well plates. Each well was supplemented with 10 μl of CCK-8 solution (Dojindo, Japan) and the 96-well plates were then returned to an incubator for 1 h. A microreader was applied to measure the OD value at 450 nm. The proliferative activity of each group of cells was determined after 0, 24, 48, 72, and 96 h of culture.

### Colony formation assay

The same number of transfected PC cells were seeded into six-well plates, the medium was changed every 2 days, and cells were allowed to grow for 1 to 2 weeks. Subsequently, the six-well plates were cleaned, fixed and dyed, and finally photographed.

### Wound healing assay

While waiting for the six-well plate to be filled with transfected cells, a wound was made in the cell layer using a sterile plastic tool; then, the wells were refreshed with medium and cells were incubated for 24 h. An Olympus BX51 microscope was used to photograph the cell layers.

### Western blot analysis

The same amount of cell protein lysate was separated by 10% sodium dodecyl sulfate (SDS) polyacrylamide gel electrophoresis and transferred to polyvinylidene fluoride (PVDF) membranes. The PVDF membranes were blocked with skim milk (5%) and then treated with specific primary antibody: RAF1, p-RAF1, ERK, p-ERK (all at 1:500; CST, USA), MMP2, MMP9, and GAPDH (all at 1:1000; Abcam, USA). Subsequently, incubation with horseradish peroxidase (HRP)-conjugated secondary antibodies was performed. Finally, EasyBlot ECL kit (Sangon, China) was used for visualizing the protein bands.

### Tumorigenicity assay in nude mice

A subcutaneous transplantation model was applied to determine the tumorigenicity *in* *vivo*. Transfected cells were collected and put in a suspension adjusted to a density of 1×10^6^ cells, which was subcutaneously injected into the right armpit of mice under pentobarbital sodium anesthesia. The weight and subcutaneous tumor volume of the mice were recorded weekly. After 8 weeks of feeding, the mice were sacrificed painlessly. Subcutaneous tumors of the nude mice were dissected and measured, then fixed with 4% paraformaldehyde and sectioned, and finally subjected to immunohistochemical detection. This study was approved by the Animal Research Ethics Committees at West China Hospital of Sichuan University, Chengdu, China.

### Immunohistochemistry (IHC)

The tumor tissues of mice were made into paraffin blocks and cut into 4-μm-thick sections. Sample sections were probed by Ki67 and PCNA primary antibodies, followed by incubation with horseradish peroxidase-conjugated secondary antibody. DAB was used as a chromogen to visualize Ki-67- and PCNA-positive staining.

### Statistical analysis

Data from at least three experiments are presented as mean ± standard deviation. Statistical analysis was performed using the two-tailed Student’s *t*-test or one-way analysis of variance (ANOVA). The correlation between LINC01559 expression and clinical pathological features was assessed via Fisher’s exact test or the Kruskal–Wallis test. SPSS 21.0 software was used for the statistical analysis. *P* < 0.05 was considered statistically significant.

### Ethics approval

The study was approved by the Human Research Ethics Committees of the First People’s Hospital of Neijiang, Neijiang, Sichuan

## Supplementary Material

Supplementary Table 1
