# Supplementary material for: Long noncoding RNA LINC01559 promotes pancreatic cancer progression by acting as a competing endogenous RNA of miR-1343-3p to upregulate RAF1 expression
Source: Aging (Albany NY). 2020 Jul 17;12(14):14452–66. doi: 10.18632/aging.103487 (PMC7425501; doi:10.18632/aging.103487)
Supplement: Supplementary Table 1 [file aging-12-103487-s001..pdf]

SUPPLEMENTARY TABLE

Supplementary Table 1. The primer sequences in this study.

Gene		Sequence
GAPDH	Forward	5′-GGAGCGAGATCCCTCCAAAAT-3′
	Reverse	5′-GGCTGTTGTCATACTTCTCATGG-3′
U6	Forward	5′-TGCGGGTGCTCGCTTCGGC-3′
	Reverse	5′-CCAGTGCAGGGTCCGAGGT-3′
LINC01559	Forward	5′-GTCCTGCAGAACTCCCTCTT-3′
	Reverse	5′-AGTCCTGGAGCTGCAGAAAT -3′
miR-1343-3p	Forward	5′-CTAGTGCAGTTGTGACTCTACCCAGGAAA-3′
	Reverse	5′-AGCTTTTCCTGGGTAGAGTCACAACTGCA-3′
RAF1	Forward	5′-GGGAGCTTGGAAGACGATCAG-3′
	Reverse	5′-ACACGGATAGTGTTGCTTGTC-3′
